# Overexpression of the clock gene Per2 suppresses oral squamous cell carcinoma progression by activating autophagy via the PI3K/AKT/mTOR pathway

**DOI:** 10.7150/jca.42771

**Published:** 2020-03-26

**Authors:** Huan Liu, Xiaobao Gong, Kai Yang

**Affiliations:** Department of Oral and Maxillofacial Surgery, The First Affiliated Hospital of Chongqing Medical University, 1 Youyi Road, Yuzhong District, Chongqing 400016, People's Republic of China

**Keywords:** clock genes, oral cancer, autophagy, carcinogenesis, Period 2

## Abstract

The current studies reveal that the clock gene Per2 is expressed at lower levels in a variety of tumors and plays a significant tumor suppressor role. However, the biological functions and mechanism of Per2 in OSCC (OSCC: oral squamous cell carcinoma) remain unclear. In this study, OSCC cells with stable overexpression or silencing of Per2 were established to explore their biological functions and mechanism *in vivo* and *in vitro*. We discovered that the expression of Per2 decreases in OSCC cells. Overexpression of Per2 promoted autophagy and apoptosis in OSCC cells and inhibited proliferation. The opposite results were obtained in Per2-silenced OSCC cells. In Per2-overexpressing OSCC cells, the expression levels of PIK3CA, p-AKT, p-mTOR, p62 and Beclin1 were significantly reduced and the LC3B II/I ratio was significantly increased. In contrast, in Per2-silenced OSCC cells, the expression levels of PIK3CA, p-AKT, p-mTOR, p62 and Beclin1 were significantly enhanced and the LC3B II/I ratio was significantly reduced. When the AKT activator SC79 was added to Per2-overexpressing OSCC cells, the increased autophagy, apoptosis and decreased proliferation were significantly rescued. Furthermore, when autophinib, an autophagy inhibitor, was added to Per2-overexpressing OSCC cells, the decreased proliferation and increased apoptosis were significantly restored. An *in vivo* tumorigenesis assay also confirmed that overexpression of Per2 suppresses the growth of OSCC. In conclusion, our research results demonstrate that Per2 suppresses OSCC progression by motivating autophagy, as well as inhibiting cell proliferation and promoting apoptosis, which were mediated by autophagy, in a PI3K/AKT/mTOR pathway-dependent manner. Per2 could potentially be used as a valuable therapeutic marker for OSCC.

## Introduction

OSCC (OSCC: oral squamous cell carcinoma) is the most common malignant tumor of the head and neck 1, 2. Currently, the conventional treatment of OSCC is surgical treatment, adjuvant radiotherapy and chemotherapy 3, 4. Although these therapy techniques have been greatly improved, the 5-year survival rate of patients with OSCC has remained at approximately 50% over the last three decades and has not been dramatically improved 4. Therefore, finding effective therapeutic targets for OSCC is of great significance for improving the survival rate of patients with OSCC.

Current studies have found that clock genes exist in almost all human cells^5^. Clock genes Per (Per1, Per2 and Per3), Cry (Cry1, Cry2), Bmal1 and Clock constitute a negative feedback loop at the transcription and translation levels, and therefore physiological activities exhibit a rhythmic oscillation at a 24h cycle, known as a circadian rhythm^6^. In the feedback loop, CLOCK/BMAL1 heterodimers, synthesized from CLOCK and BMAL1, activates the gene transcription of Pers (Per1, 2, 3) and Crys (Cry1, 2). The translated PERs and CRYs forms heterodimers and performs nuclear translocation, which result in the feedback inhibition of CLCOK/BMAL1 and the reduce in their transcriptional activity^5^, ^7^, ^8^. Per2 is an important negative regulator in the feedback loop^7^, ^9^, ^10^. Current studies have shown that clock genes regulate numerous physiological activities, including cell proliferation and apoptosis 11-14. Abnormal expression of the clock gene is strongly linked to the occurrence and development of various diseases, such as tumors 15, 16. Per2 is an important core clock gene 6, 15, and existing studies have reported that Per2 expression is strikingly lower in a number of human cancers, such as gastric cancer and non-small cell lung cancer, and closely related to the development and progression of cancer 17-20. Xiong H et al found that Per2 is expressed at lower levels in OSCC tissues and is significantly associated with the clinical stage and survival time of patients 21. These reports suggest that Per2 is a crucial tumor suppressor gene, but its specific mechanism of tumor suppression is unknown.

In this study, we corroborated for the first time that, by establishing stable Per2-overexpressing and Per2-silenced OSCC cells, Per2 regulates cell autophagy dependent on the PI3K/AKT/mTOR pathway in OSCC cells, and it was found that autophagy induces the increase of apoptosis and the decrease of proliferation. *In vivo* experiments have further indicated that Per2 overexpression inhibits OSCC growth.

## Materials and Methods

### Cell culture and reagents

The normal oral mucosa HOMEC cells used in our studies were purchased from Shanghai Bioleaf Biotechnology Co., Ltd. (Shanghai, China). Oral squamous cell carcinoma TSCCA cells were purchased from Shanghai Zhong Qiao Xin Zhou Biotechnology Co., Ltd. (Shanghai, China). Oral squamous cell carcinoma SCC15 and CAL27 cells were donated by Professor Huang Enyi from Chongqing Key Laboratory of Oral Diseases and Biomedicine. The cells were cultured in DMEM (Gibco, USA) containing 10% fetal bovine serum and 1% penicillin-streptomycin in a 37°C incubator containing 5% CO_2_. The cells were subcultured or their culture medium was replaced every 2~3 days.

### Per2 overexpression and knockdown lentivirus vector construction and packing

To construct a Per2-overexpressing lentivirus vector, the primers were designed by adding a homologous recombination sequence at the 5' end for acquiring the Per2 gene. The primer sequence are as follows: Per2-P1:AGGTCGACTCTAGAGGATCCCGCCATGAATGGATACGCGGAATTTCGCCCAG;Per2-P2:TCCTTGTAGTCCATACCCGTCTGCTCTTCGATCCTGTGATTCAAG. PCR was performed to obtain Per2 amplification products. Then, the amplification products and linearized vector, which was restriction enzyme-digested by Age I, were subjected to a recombination reaction. The lentivirus plasmid was transformed into competent E. coli DH5α and cultured on an LB plate containing aminonucleoside antibiotics. The monoclonal colony was picked and inoculated in LB medium for shaking overnight. The plasmid DNA was extracted according to the Qiagen plasmid extraction protocol, followed by sequencing and sequence alignment analysis. 293T cells were seeded in culture dishes and cultured to a density of 70% to 80%, and each DNA solution, including the GV vector plasmid, pHelper1.0 vector plasmid, and pHelper2.0 vector plasmid, was cotransfected with Genechem transfection reagent for 48 hours. The supernatant of 293T cells was collected, filtered and stored properly.

In order to knock down Per2 expression, according to the human Per2 mRNA sequence (Gene ID: NM_022817), three sequences for different targets of Per2 were designed by siRNA design software to synthesize shRNA-I~III. Per2-shRNA-I: 5'-CCGGGCCAGAGTCCAGATACCTTTACTCGAGTAAAGGTATCTGGACTCTGGCTTTTTG-3';Per2-shRNA-II:5'-CCGGGCATCCATATTTCACTGTAAACTCGAGTTTACAGTGAAATATGGATGCTTTTTG-3';Per2-shRNA-III:5'-CCGGGCACACACACAAAGAACTGATACTCGAGTATCAGTTCTTTGTGTGTGTCTTTTTG-3'. Then, the Age I/EcoR I double-digested vector and the double-stranded DNA were ligated with T4 DNA ligase to construct the shRNA-I~III lentivirus plasmid, and the interference-free scramble plasmid (sequence: TTCTCCGAACGTGTCACGT) was constructed as a negative control. The following steps were identical to the Per2-overexpression plasmid to construct the Per2-knockdown lentivirus.

### Per2-overexpression and Per2-knockdown lentivirus infection

OSCC cells were prepared as a suspension and inoculated in a six-well plate with 8×10^4^ cells every well for 16-24 hours. When cell fusion reached 20~ 30%, 40 μl of HitransG P infection enhancing solution was added, as well as suitable volumes of lentivirus infection reagent according to an MOI = 50. After 24 hours, the medium was replaced with fresh complete medium. At 72 hours after transfection, fluorescence expression was observed by fluorescence microscopy (Eclipse Ti, NIKON, Japan). Then, the transfected cells were subcultured in medium containing 2 μg/ml puromycin. The concentration of puromycin was changed to 1 μg/ml to ensure Per2 expression seven days later. As described above, we obtained stable Per2-OE cells (Per2-OE cells: Per2-overexpressing TSCCA cells) and Per2-RNAi cells (Per2-RNAi cells: Per2-silenced CAL27 cells). A negative control lentivirus, constructed by an empty vector in which the Per2 gene sequence was not inserted, was transfected into TSCCA cells as a negative control for the Per2-OE group, namely NC group. Another control lentivirus, whose vector was inserted with a widely-recognized scramble sequence (TTCTCCGAACGTGTCACGT), was transfected into CAL27 cells as a negative control for the Per2-RNAi group, namely scramble group. Untreated TSCCA and CAL27 cells served as blank control groups (Blank).

### CCK-8

Following the CCK reagent protocol (Cell Counting Kit-8, Dojindo, Japan), the cells were inoculated onto a 96-well plate at 1000 cells/100 μl per well. Each group had 3 replicate wells and grew for one night. Then, 10 μl of CCK-8 solution was added to each well at 0, 24, 48, 72, and 96 hours. After addition for 2 hours each time, the OD value at 450 nm was detected by a microplate reader (Gene Company Limited, Hong Kong, China). The growth curves were drawn with the time and the corresponding OD values.

### Immunofluorescence assay

The cells were seeded on slides in a six-well plate and adhered. The sample was fixed in cold methanol at -20°C for 25 minutes and washed with TBST three times. It was subjected to immunostaining blocking solution (P0260, Beyotime, China) for 10 minutes. The LC3B (ab192890, Abcam, UK) or p62 (ab109012, Abcam, UK) antibody was incubated separately overnight at 4°C, followed by Cy3-labeled goat anti-rabbit IgG (H+L, A23320, Abbkine, China) for 1 hour in the dark at room temperature. Finally, the slides were treated with DAPI solution and antifluorescence quencher. The slides were detected using a fluorescence microscope (BX51TRF, Olympus, Japan). Five fields were randomly chosen in every sample, and the fluorescence intensity was analyzed using ImageJ-5.0 software (Windows, 64-bit Java 1.8.0_112).

### Transmission electron microscopy (TEM)

The cells were digested and centrifuged at 1200 r/min for 10 minutes, fixed overnight at 4°C with 4% glutaraldehyde, and fixed with 2.5% osmium tetroxide. The cells were dehydrated in ethanol with a gradient-increasing concentration and then embedded in paraffin. The samples were cut into 80-nm sections. The morphology and number of autophagosomes in the sections were observed with a transmission electron microscope (Hitachi-7500, Hitachi Limited, Japan). Ten cells were selected at random in each group and used in the following formula: autophagosome density = autophagosome number / cell number.

### Flow cytometry

#### Cell proliferation assay

The cells were collected in the logarithmic growth phase and washed twice with PBS. They were resuspended in 100 μl of PBS and 500 μl of cold 75% ethanol overnight at 4°C. Then, they were washed twice with PBS, and 400 μl of propidium iodide solution and 100 μl of RNase (100 μg/ml) were added, followed by mixing and incubating for 30 minutes at 4°C in the dark. Next, the cells were counted by flow cytometry (FACSVantage SE, BD Biosciences, USA). A total of 2~3 million cells were counted, and the results were analyzed by the cell cycle fitting software ModFit. The cell proliferation index was determined according to the following formula: (S + G2 / M) / (G0 / G1 + S + G2 / M) ×100%.

#### Apoptosis detection

The cells were collected, and the concentration was adjusted to 1×10^6^ cells/ml with PBS. Then, 200 μl of Annexin V-APC staining solution was added to 1 ml of cell suspension and protected from light for 15 minutes at room temperature. Next, 200 μl of propidium iodide staining solution was added and mixed. The cells were tested by flow cytometry (FACSVantage SE, BD Biosciences, USA) and counted using the following equation: apoptosis index = number of apoptosis cells / total number of detected cells ×100%.

### TUNEL analysis

The TUNEL assay was performed according to the instructions of the Apoptosis Detection Kit (TUNEL-POD, Mengbio, China). The cells were seeded on slides that were placed in a 6-well plate. The slides were immersed in 4% paraformaldehyde solution and PBS separately for 15 minutes at room temperature. Next, each sample was incubated with 100 μl of proteinase K solution (20 μg/ml) for 5 minutes and washed with PBS. A 50-μl TdT-labeled reaction mixture was added to each sample (the mixture was compounded: biotin-labeled reactant: TdT enzyme (25×) = 48 μl: 2 μl). The slides were covered with parafilm and placed in a wet box for 60 minutes at 37°C. The slides were rinsed with PBS and deionized water at room temperature. Then, POD diluent was diluted 1:25, and each sample was treated with 50 μl of diluted solution at 37°C for 30 minutes. Finally, the slides were observed by fluorescence microscopy (BX51TRF, OLYMPUS, Japan). The experiment was repeated three times, and five different fields were randomly selected. The numbers of cells were calculated using ImageJ-5.0 software (Windows, 64-bit Java 1.8.0_112). The TUNEL-positive rate was equal to the number of TUNEL-positive cells divided by the total cell number × 100%.

### Real-time quantitative polymerase chain reaction (RT-qPCR)

Total RNA was extracted from each group of cells according to the TaKaRa RNAiso Plus (9180, TaKaRa Bio, China) instructions. Total RNA was dissolved in DEPC water, and its OA value at 260 nm and 280 nm was measured by NanoDrop ND2000 (Thermo Scientific). The RNA purity and concentration ​​were sequentially calculated. Complementary DNA (cDNA) was reverse transcribed using the PrimeScript™ RT Reagent Kit with gDNA Eraser (Perfect Real Time) (RR047A, TaKaRa Bio, China). The reaction system was 10 μl, and the reaction conditions were 37°C for 15 minutes, followed by 85°C for 5 s and 4°C for 4 minutes. The primers for the target genes (Per2 and GAPDH) were designed using Oligo7.0 software *(The sequences are listed in Supplementary Table S1)*. PCR amplification was conducted using a C-1000^TM^ Thermal Cycler (Bio-Rad, CA, USA). Real-time PCR was performed using TB Green^®^ Premix Ex Taq™ II (Tli RNaseH Plus) (RR820A, TaKaRa Bio, China) according to the manufacturer's instructions. The reaction conditions were 40 cycles of 95°C for 90 s and 95°C for 10 s and 60°C for 30 s. The relative expression level of the desired gene was analyzed using the 2^-ΔΔCt^ method.

### Western blotting

Cells were lysed for 30 minutes in cold RIPA lysis buffer (P0013B, Beyotime, China) containing PMSF (ST506, Beyotime, China) and phosphatase inhibitor cocktail A (P1080, Beyotime, China). The supernatant was collected after centrifugation at 12000*g* for 15 minutes at 4°C. The protein concentration was measured using a BCA Protein Quantitation Kit (P0010, Beyotime, China). Protein (30~50 μg) was separated by 8%~15% SDS-PAGE gel electrophoresis and then transferred onto a 0.22-μm PVDF membrane. Later, the membrane was blocked for 1 hour with 5% skim milk and incubated with primary antibodies against PER2, PIK3CA, AKT, p-AKT, mTOR, p-mTOR, LC3B, p62, Beclin1 and GAPDH at 4°C for 14~16 hours *(Information about the antibodies is provided in Supplementary Table S2).* The PVDF membrane was immersed in HRP-labeled secondary antibody dilutant and shaken gently for 1 hour at room temperature. A hypersensitive chemiluminescent substrate (34577, Thermo Scientific, USA) was applied to the membrane, and its protein band was analyzed in an ECL-Advance Western Blotting Imaging System (ChemiDoc XRS+, Bio-Rad, USA). The intensities of total AKT and total-mTOR were used as a control for p-AKT or p-mTOR separately, and the intensities of GAPDH were used as a control for all the other bands. Each test was conducted on the same membrane and repeated 3 times. Data were analyzed by ImageJ-5.0 software (Windows, 64-bit Java 1.8.0_112).

### *In vivo* tumorigenesis experiments

Six BALB/c nu/nu nude mice (SPF, 18~22 g, 4 weeks old) were purchased and fed in Chongqing Medical University Laboratory Animal Center. They were randomly divided into the Per2-OE group and NC group, with 3 mice in each group. Each mouse was given a subcutaneous injection into the left back with 200 μl of a suspension containing 1 × 10^7^ Per2-OE or NC TSCCA cells. Tumor size was measured using calipers every 4 days after injection. After 4 weeks, tumor formation was obvious, and the mice were sacrificed by cervical dislocation. The tumor was isolated, and its weight and volume were recorded (tumor volume = 1/2 × length × width^2^). All animal experimental procedures were approved by the Laboratory Animal Use Management Committee of the Experimental Animal Research Institute of Chongqing Medical University (approval number: 2018-102).

### Statistical analysis

All statistical analyses were performed by GraphPad Prism 8.0 (GraphPad Software, La Jolla, CA) or SPSS 23 (IBM Corporation, USA). The student's t test was used to analyze differences between two independent groups. One-way analysis of variance (ANOVA) was used for comparisons between multiple groups. Data are shown as the mean±SD from at least three independent experiments. Values of *P* < 0.05 were considered statistically significant.

## Results

### Per2 expression is decreased in OSCC cell lines

**Figure 1:**
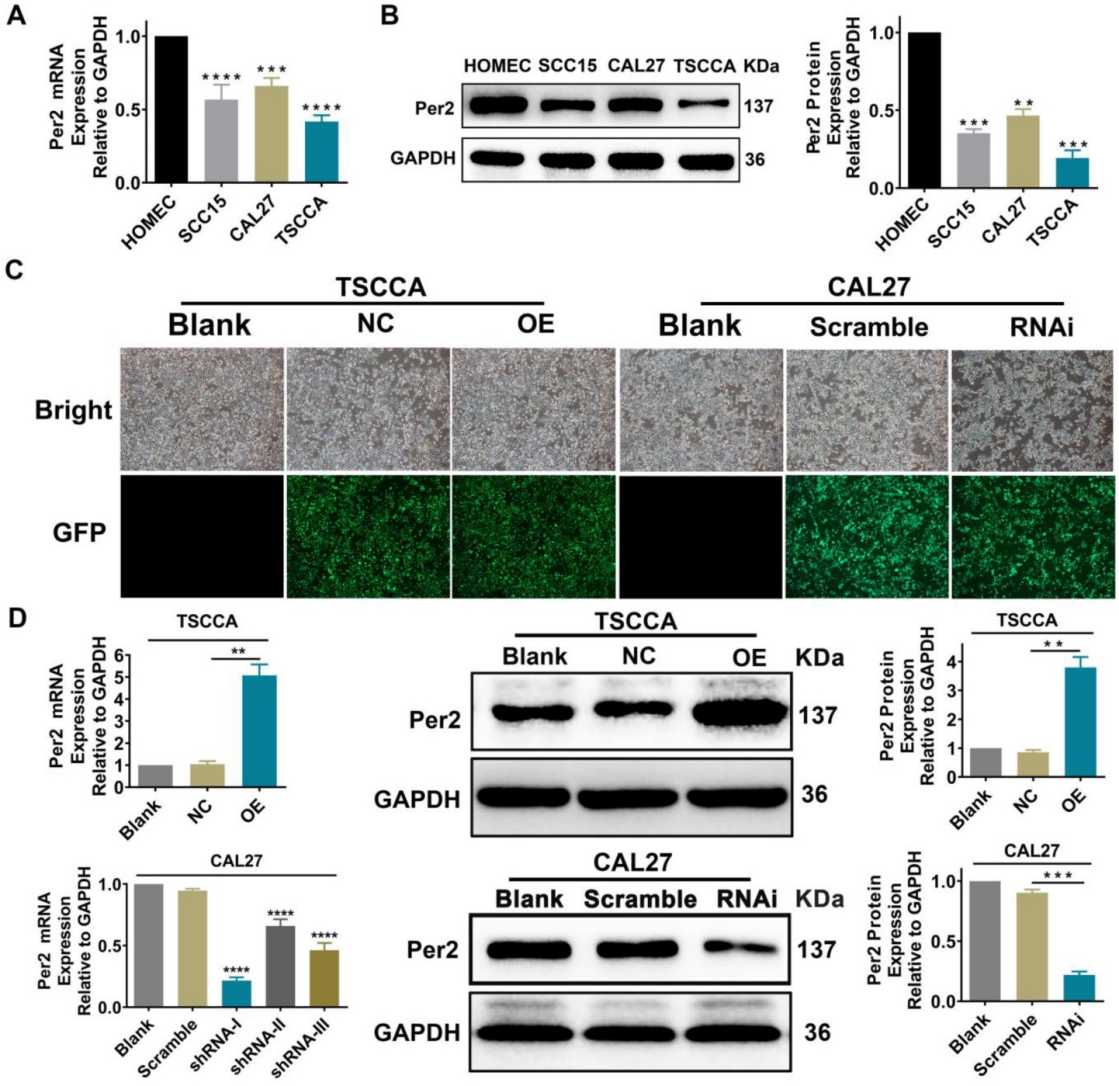
** Per2 expression is decreased in OSCC cells. Per2 was successfully overexpressed and knocked down separately in TSCCA cells and CAL27 cells. (A)** RT-qPCR results indicated that Per2 mRNA expression in TSCCA, SCC15 and CAL27 cells was markedly lower than that in HOMEC cells. **(B)** Western blotting results indicated that Per2 protein expression in TSCCA, SCC15 and CAL27 cells was markedly lower than that in HOMEC cells. **(C)** The green fluorescent light densities indicated that the lentivirus plasmid was highly effectively integrated into the genome of TSCCA and CAL27 cells. **(D)** Verification of the knockdown efficiency of three shRNAs (shRNA-I, shRNA-II, and shRNA-III) targeting different sites of Per2 in CAL27 cells verified that shRNA-I was the most effective shRNA for knockdown of Per2 in CAL27 cells. All data represent three independent experiments. Data are shown as the mean ± SD (n ≥ 3). *P < 0.05, **P < 0.01, ***P < 0.001, ****P < 0.0001.

Per2 mRNA and protein expression levels were detected by RT-qPCR and western blotting in normal oral mucosal HOMEC cells and oral squamous cell carcinoma TSCCA, SCC15 and CAL27 cells. The results verified that the Per2 mRNA and protein expression levels of three OSCC cells were obviously lower than those of HOMEC cells (*P* < 0.01) (Figure 1A, 1 B).

### Stable overexpression or silencing of Per2 OSCC cells was established

TSCCA cells with the lowest Per2 expression were chosen to establish stable Per2-OE cells, and the overexpression efficiency of Per2-OE cells was 2.94±0.29 fold. Among the three Per2-knockdown plasmids shRNA-Ⅰ~Ⅲ, shRNA-I was the most effective at knocking down Per2 expression (*P* < 0.001) and was therefore transfected into CAL27 cells whose Per2 expression was the highest in comparison with the other two cell lines. Stable Per2-RNAi cells were constructed, and the interference efficiency was 68.4%±2.5% (Figure 1C,1D).

### Overexpression of Per2 inhibits tumor cell proliferation and promotes cell apoptosis; knockdown of Per2 promotes proliferation and inhibits apoptosis

**Figure 2:**
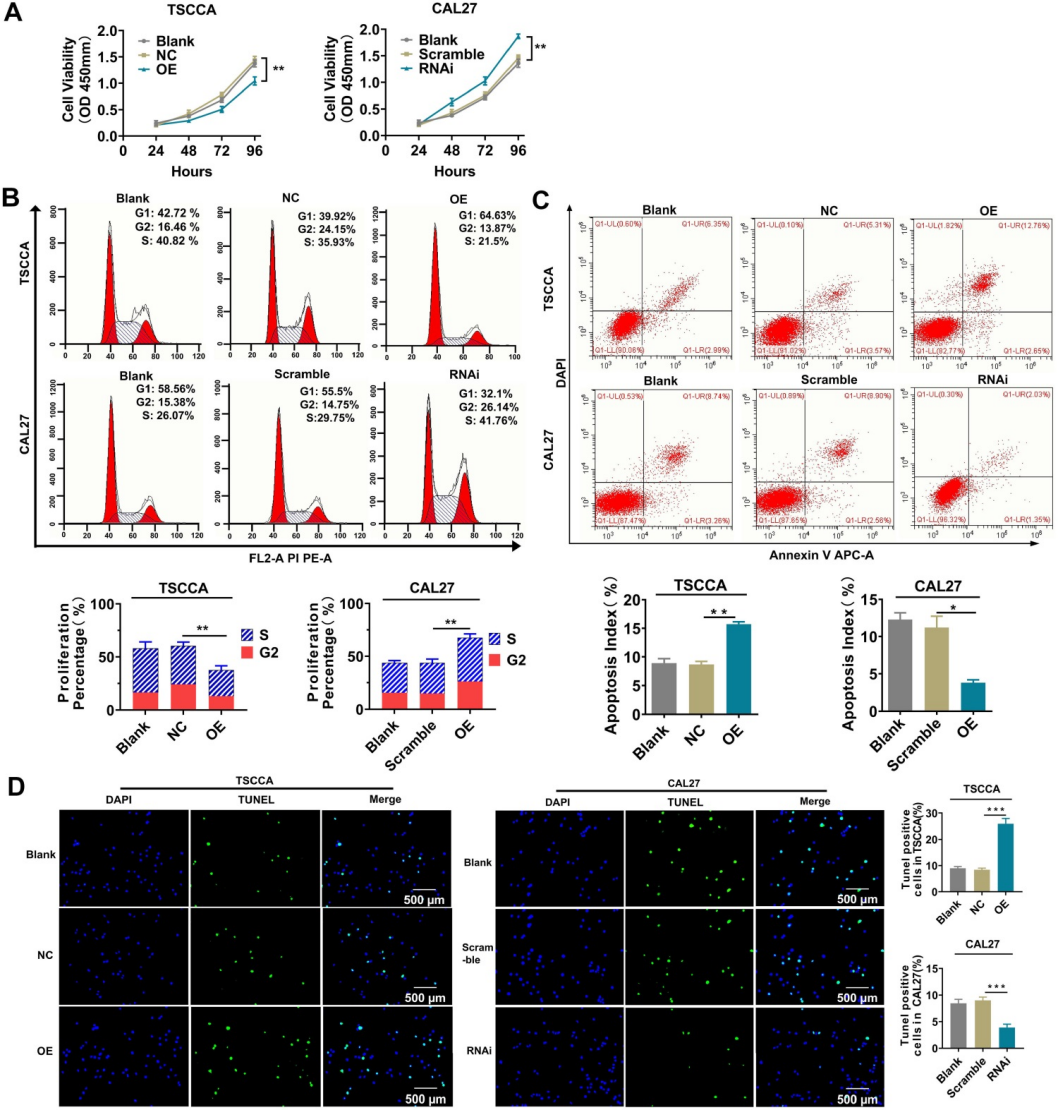
** Per2 inhibits the proliferation and promotes apoptosis of OSCC cells, whereas Per2 depletion facilitates the proliferation and suppresses apoptosis of OSCC cells. (A)** The CCK-8 assay showed that the proliferation of TSCCA was significantly decreased when Per2 was overexpressed, and the proliferation of Per2-knockdown CAL27 cells was significantly increased. **(B)** Flow cytometry analysis revealed that the proliferation percentage of Per2-OE cells was notably reduced and that of Per2-RNAi cells increased. **(C)** Flow cytometry analysis revealed that the apoptosis index of the Per2-OE group decreased significantly, and it increased in the Per2-RNAi group. **(D)** The TUNEL assay showed that the rate of TUNEL-positive cells was elevated and decreased significantly following Per2 overexpression in TSCCA cells or Per2 silencing in CAL27 cells. All data represent three independent experiments. Data are presented as the mean ± SD (n ≥ 3). *P < 0.05; **P < 0.01; ***P < 0.001; ****P < 0.0001.

The cell proliferation and apoptosis phenotypes were detected by flow cytometry, CCK-8 and TUNEL experiments to investigate the influences of altered Per2 expression on the proliferation and apoptosis of OSCC cells. The results of the CCK-8 assay and flow cytometry showed that the proliferation of TSCCA cells was significantly decreased after overexpression of Per2 (*P* < 0.01), and it was significantly improved in Per2-RNAi cells (*P* < 0.01) (Figure 2A, 2B). A flow cytometry apoptosis assay indicated that the apoptosis index was upregulated in Per2-OE cells (*P* < 0.01) and was significantly decreased in Per2-RNAi cells (*P* < 0.05) (Figure 2C). TUNEL analysis showed that the proportion of TUNEL-positive cells in Per2-OE cells was significantly elevated, and the numbers of TUNEL-positive cells in the Per2-RNAi group were significantly reduced (*P* < 0.001) (Figure 2D).

### Overexpression of Per2 activates autophagy while knockdown of Per2 inhibits autophagy in OSCC cells

**Figure 3:**
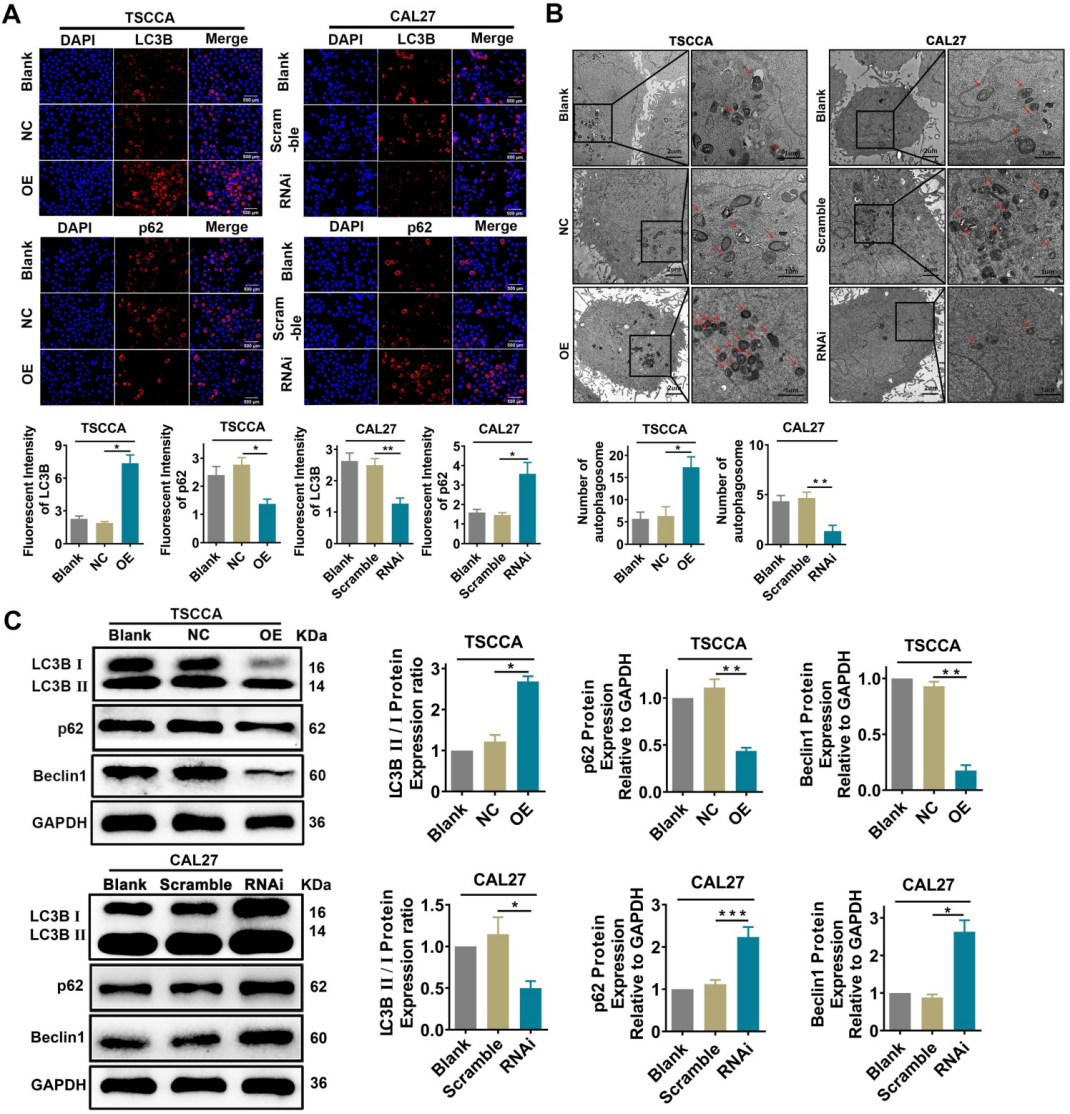
** Overexpression of Per2 promotes autophagy in OSCC cells, and knockdown of Per2 inhibits autophagy in OSCC cells. (A)** The immunofluorescence assay showed that the fluorescence intensity of LC3B was increased significantly in Per2-OE cells, and the fluorescence intensity of p62 was reduced (scale bars=500 µm). Instead, the results were both opposite in Per2-RNAi cells; the fluorescence intensity of LC3B and p62 were obviously decreased and increased, respectively. **(B)** The autophagosomes had a higher density in Per2-OE cells as observed by TEM, and there were fewer autophagosomes in CAL27 cells when Per2 was knocked down (low magnification scale bars = 2 µm; high magnification scale bars = 1 µm). **(C)** When detected by western blotting, the LC3B II/I ratio was enhanced after overexpression of Per2 in TSCCA cells, and the expression levels of p62 and Beclin1 protein decreased significantly. The LC3B II/I ratio was reduced and p62 and Beclin1 protein levels were higher after Per2 knockdown. All data represent three independent experiments. Data are presented as the mean ± SD (n ≥ 3). *P < 0.05; **P < 0.01; ***P < 0.001; ****P < 0.0001.

Immunofluorescence analysis showed that the fluorescence intensity of LC3B protein was significantly enhanced in Per2-OE cells (*P* < 0.05), while the fluorescence intensity of p62 protein was significantly reduced (*P* < 0.05). In contrast, the fluorescence intensity of the LC3B protein was attenuated (*P* < 0.01) in Per2-silenced CAL27 cells, and the fluorescence intensity of p62 protein was significantly upregulated (*P* < 0.05) (Figure 3A). According to the transmission electron microscopy analysis (Figure 3B), the autophagic density was visibly elevated after overexpression of Per2 (*P* < 0.05), and it was significantly lessened after silencing of Per2 (*P* < 0.01). In addition, as detected by western blotting, the ratio of LC3B II/I was significantly increased in the Per2-OE group (*P* < 0.05), and the expression levels of p62 and Beclin1 were notably decreased (*P* < 0.01) (Figure 3C). In contrast, the LC3B II/I ratio was significantly decreased (*P* < 0.05) in the Per2-RNAi group, and the p62 and Beclin1 expression levels were significantly elevated (*P* < 0.05) (Figure 3C).

### Per2 regulates the PI3K/AKT/mTOR signaling pathway in OSCC cells

**Figure 4:**
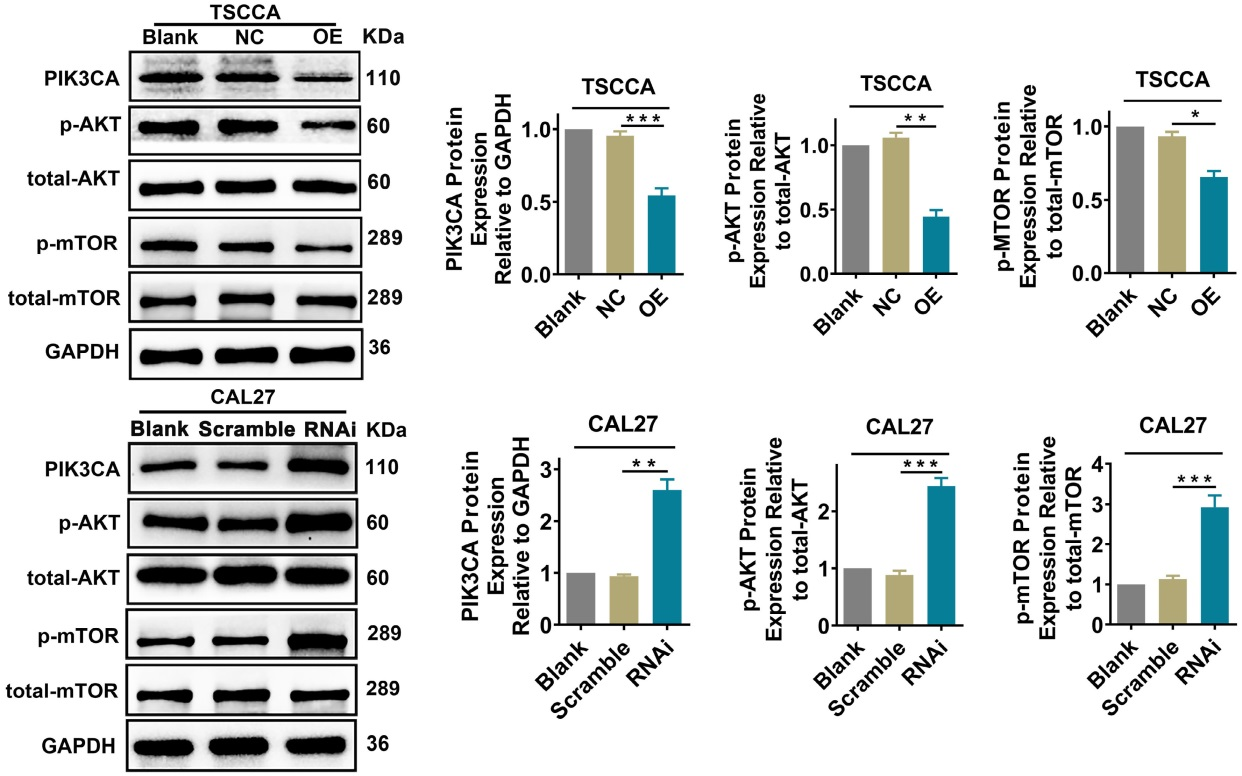
** Per2 overexpression prevents the activation of the PI3K/AKT/mTOR pathway, and Per2 silencing contributes to activating the PI3K/AKT/mTOR pathway.** The protein expression levels of PIK3CA, p-AKT and p-mTOR in Per2-OE cells were notably downregulated as detected by western blotting, and the levels of total AKT and total mTOR were not evidently changed. In Per2-RNAi cells, the PIK3CA, p-AKT and p-mTOR protein levels were obviously upregulated, and there were also no alterations in total AKT and total mTOR expression. All data represent three independent experiments. Data are presented as the mean ± SD (n ≥ 3). *P < 0.05; **P < 0.01; ***P <0.001; ****P < 0.0001.

To explore whether Per2 affects the PI3K/AKT/mTOR signaling pathway, we examined the expression of the pivotal proteins PIK3CA, p-AKT, and p-mTOR in the PI3K/AKT/mTOR pathway by western blotting. The expression of total AKT or total mTOR was not detectably changed (*P* > 0.05). Nevertheless, the expression of PIK3CA, p-AKT and p-mTOR was obviously downregulated in the Per2-OE group in contrast with the control groups (*P* < 0.05). In Per2-RNAi cells, PIK3CA, p-AKT and p-mTOR protein expression was markedly higher compared with the controls (*P* < 0.01) (Figure 4). These consequences imply that overexpression of Per2 prevents activation of the PI3K/AKT/mTOR signaling pathway; meanwhile, silencing of Per2 enhances the PI3K/AKT/mTOR signaling pathway.

### Per2 regulates autophagy, proliferation and apoptosis through the AKT/mTOR signaling pathway

**Figure 5:**
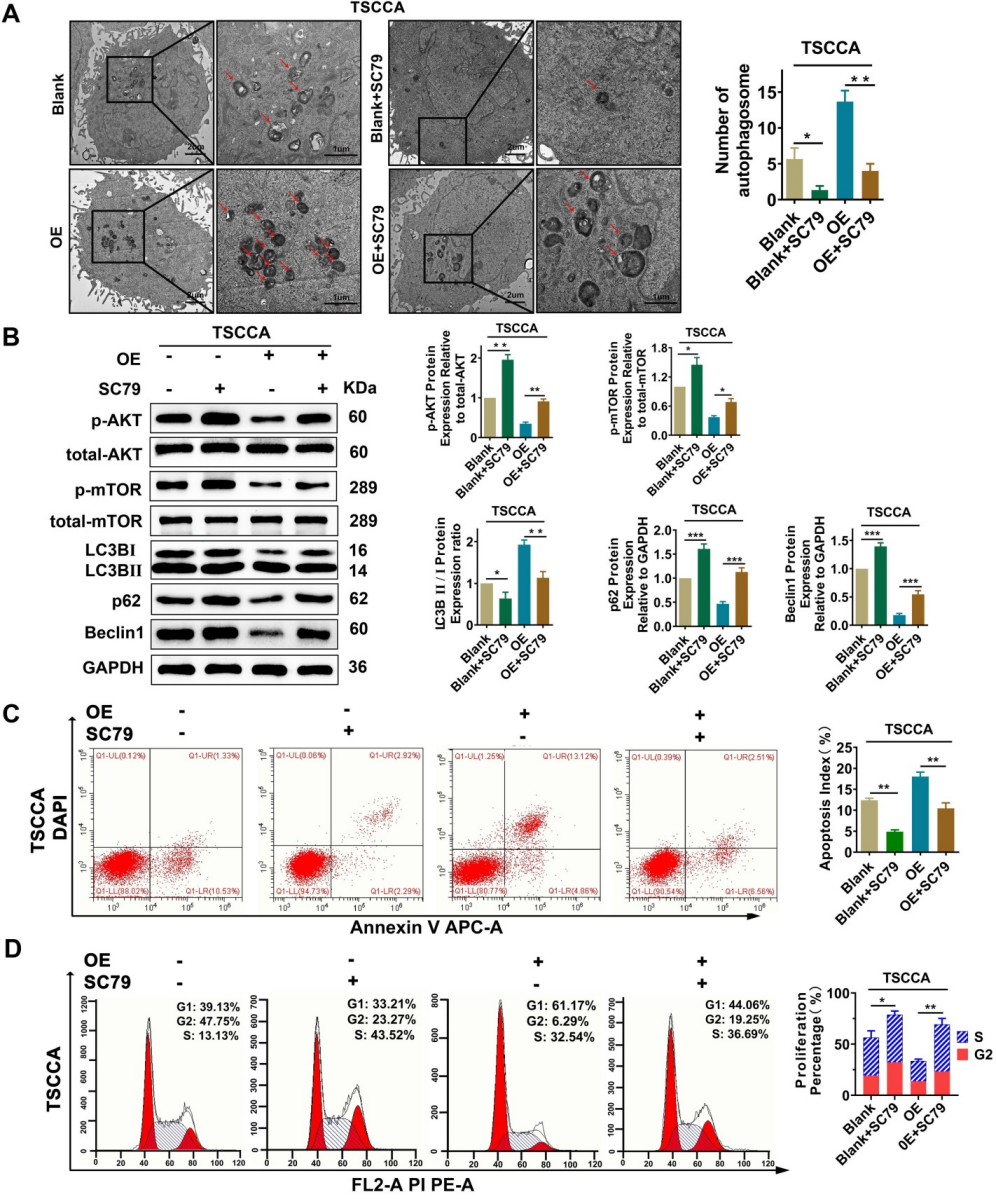
** Per2 overexpression inhibits proliferation and promotes apoptosis and autophagy in OSCC cells via the PI3K/AKT/mTOR signaling pathway. (A)** TEM experiments showed that the elevated autophagosome density was obviously decreased significantly after the addition of SC79 in Per2-OE cells (low magnification scale bars = 2 μm; high magnification scale bars = 1 μm). **(B)** Western blotting indicated that, following the addition of SC79, the level of p-mTOR was increased; moreover, the LC3B II/I ratio was significantly decreased, while p62 and Beclin1 protein expression was significantly elevated. **(C)** The apoptosis index as measured by flow cytometry was significantly reduced after the addition of SC79 to Per2-OE cells. All data represent three independent experiments. **(D)** Flow cytometry indicated that the proliferation percentage of Per2-overexpressing TSCCA cells was upregulated as a result of SC79 addition. Data are presented as the mean ± SD (n ≥ 3). *P < 0.05; **P < 0.01; ***P < 0.001; ****P < 0.0001.

To investigate whether Per2 has an impact on OSCC proliferation, autophagy, and apoptosis through the AKT/mTOR pathway, the AKT activator SC79 (HY-18749, MCE, New Jersey, USA) was added to Per2-OE cells. Then, transmission electron microscopy observation, western blotting and flow cytometry were performed. The results revealed that, compared with the control group, the increase mediated by Per2-overexpression in autophagic density, LC3B II/I ratio and apoptosis index were significantly decreased to return (*P* < 0.05) (Figure 5A, 5B, 5C) due to the addition of SC79. Meanwhile, the reduced p62 and Beclin1 protein expression levels and cell proliferation index were significantly reduced to revert (Figure 5B, 5D) (*P* < 0.01). These consequences clarified that Per2 mediates cell autophagy, proliferation and apoptosis reliant on the AKT/mTOR pathway in OSCC.

### Overexpression of Per2 induces decreased proliferation and increased apoptosis by promoting autophagy in OSCC cells

**Figure 6:**
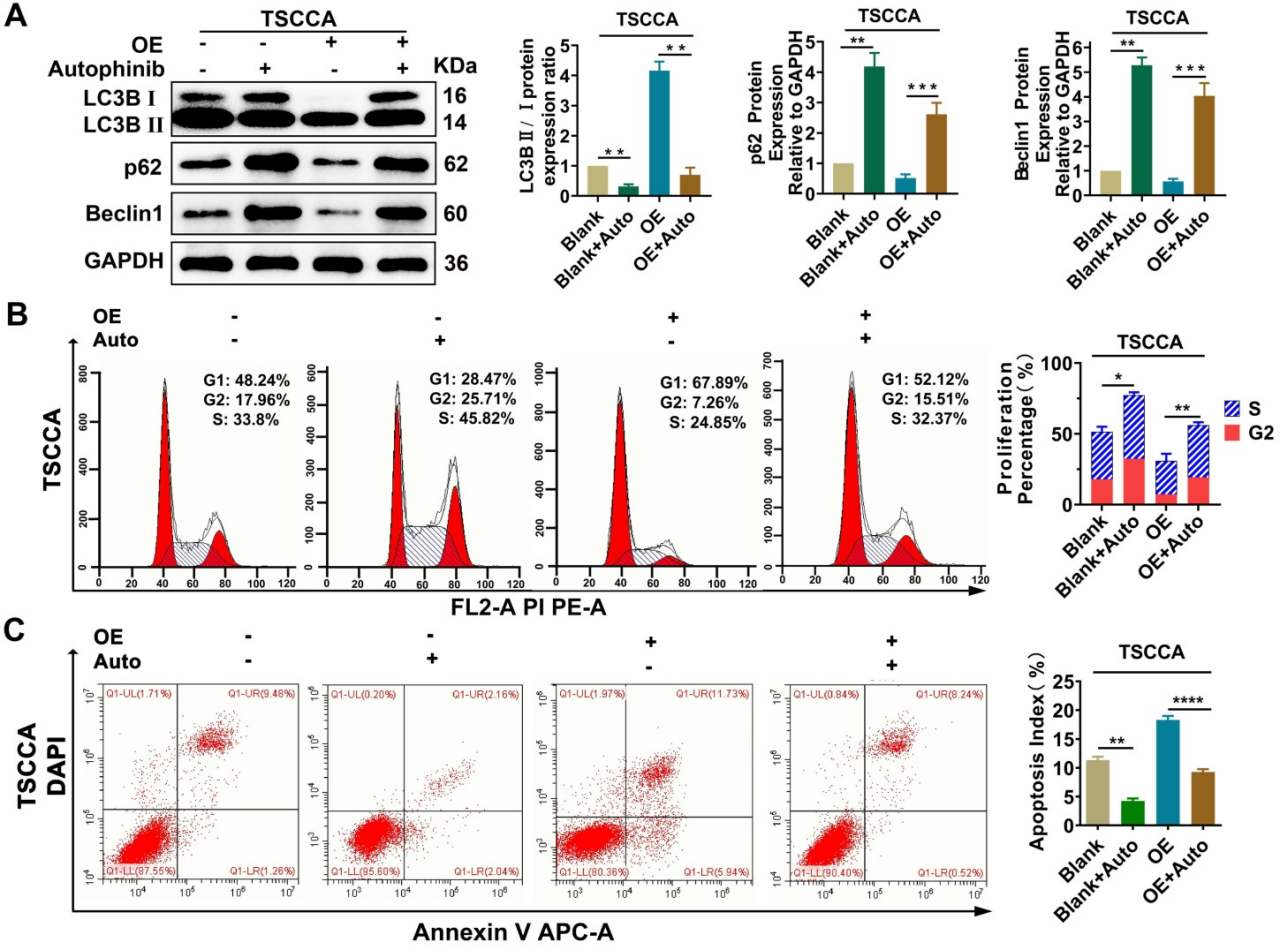
** Per2 regulates proliferation and apoptosis in OSCC cells dependent on autophagy. (A)** Western blotting indicated that the LC3B II/I ratio was markedly downregulated and p62 and Beclin1 expression were evidently increased in Per2-OE cells owing to the addition of autophinib. **(B)** Flow cytometry showed that the proliferation percentage of Per2-OE cells was obviously expanded because of autophinib. **(C)** Flow cytometry showed that the apoptotic index of Per2-OE cells was significantly decreased because of autophinib. All data represent three independent experiments. Data are presented as the mean ± SD (n ≥ 3). *P < 0.05; **P < 0.01; ***P < 0.001; ****P < 0.0001.

To explore the regulatory relationship between autophagy and proliferation, apoptosis, treatment with the autophagy inhibitor autophinib (HY101920, MCE, USA) was performed and then western blotting verified that autophagy was restrained effectively (*P* < 0.01) (Figure 6A). Flow cytometry were conducted on Per2-OE cells after adding of autophinib, compared with the controls, the reduced cell proliferation percentage and the increased apoptosis index (*P* < 0.05), induced by Per2-overexpression, were both reversed effectively (*P* < 0.001) (Figure 6B, 6C). These results suggest that the regulation of Per2 on cell proliferation and apoptosis is dependent on autophagy in OSCC cells.

### Overexpression of Per2 suppresses tumor growth *in vivo*

**Figure 7:**
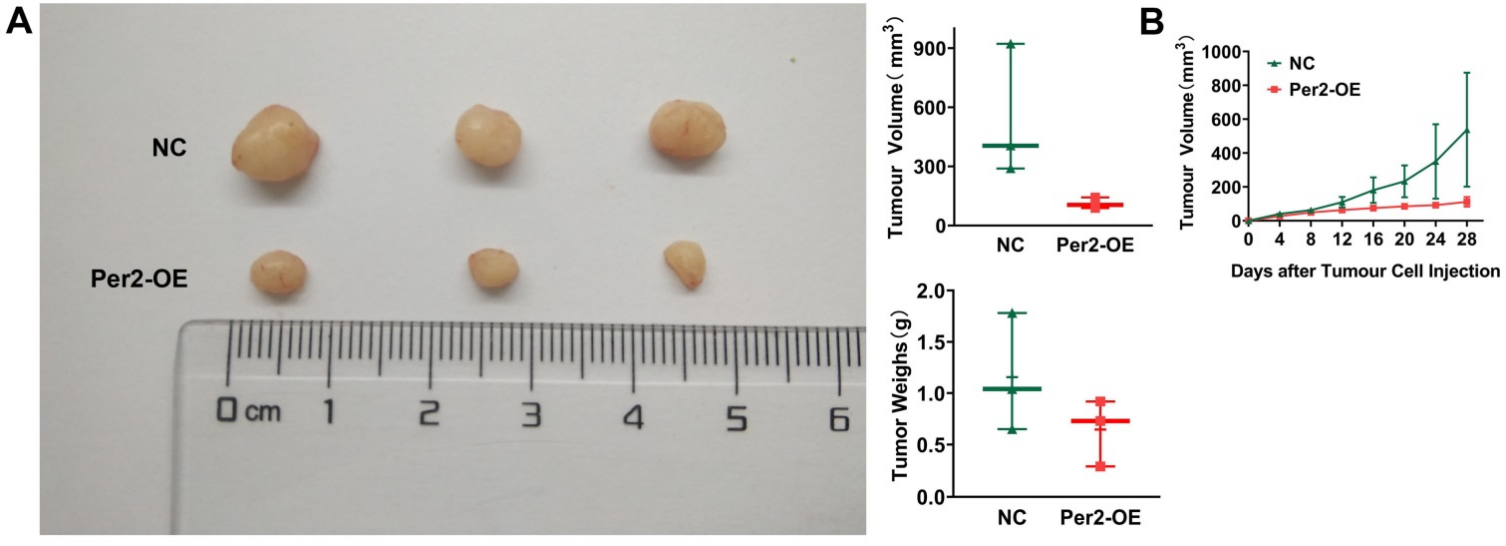
** Overexpression of Per2 suppresses tumorigenesis of OSCC *in vivo*. (A)** The tumor volume and weight of mice in the Per2-OE group were markedly lower than those in the NC group (N=3). **(B)** The growth curve of the subcutaneous tumors displayed that the tumors in the Per2-OE group grew more slowly. Data are presented as the mean ± SD (n ≥ 3). *P < 0.05; **P < 0.01; ***P < 0.001; ****P < 0.0001.

*In vivo* tumorigenesis assays in nude mice revealed that tumor weight and volume in the Per2-OE group were significantly smaller than those in the NC group (Figure 7A). As seen from the growth curve of subcutaneous tumors, tumor growth was slower in the Per2-OE group compared with that in the controls (*P* < 0.05) (Figure 7B). The result manifested that Per2 overexpression can restrain tumor growth *in vivo*.

## Discussion

A few studies have exemplified that the expression of the circadian clock gene Per2 is markedly downregulated in non-small cell lung cancer, breast cancer, ovarian cancer, gastric cancer, colorectal cancer and liver cancer 17-19, 22, 23, and it is tightly connected to the occurrence and development of cancer. Xiang et al. reported that overexpression of Per2 distinctly inhibits tumor cell growth, migration and invasion 12, 19, 24. Previous studies have also reported that overexpression of Per2 promotes apoptosis and inhibits proliferation in glioma, breast, leukemia and lung cancer cells. It is characterized by noteworthy tumor suppressor effects 11, 12, 25, 26. We also found previously that Per2 is expressed at lower levels in OSCC tissues and is markedly associated with the clinical stage and survival time of patients 21. The above studies suggest that Per2 is an important tumor suppressor gene, but its antineoplastic mechanism is ambiguous, so it is of great significance to further examine its underlying mechanism.

The PI3K/AKT/mTOR signaling pathway is one of the most frequently activated signaling pathways in tumorigenesis and progression, including OSCC 27, 28. The PI3K/AKT/mTOR pathway has an important effect on regulating autophagy, proliferation, apoptosis and so on, sequentially affecting the occurrence and development of cancer 29-32. However, it is still unclear whether Per2 affects the PI3K/AKT/mTOR pathway in OSCC. In this study, we noted that, by establishing stable OSCC cells of Per2 overexpression and Per2 knockdown, combined with functional recovery experiments, Per2 regulates autophagy, apoptosis and proliferation of OSCC cells depending on the PI3K/AKT/mTOR pathway.

Autophagy refers to the intracellular process in which autophagosomes, with a double-membraned structure, encapsulate the degradation components and transport them to the lysosome for degradation and recycling, which is a widespread life phenomenon of eukaryotic cells 33, 34. Recent studies have determined that autophagy is correlated with tumorigenesis 35, 36. Cell autophagy often has complex crosstalk with apoptosis and proliferation 37, 38. Under different cell types, diverse environments and different stimulating factors, cell autophagy can inhibit or promote apoptosis, as well as inhibit or promote cell proliferation 37, 39, 40. Therefore, autophagy appears to exert dual functions by suppressing or promoting cancer. In the present study, we found that autophagy and apoptosis were improved in Per2-overexpressing OSCC cells, and proliferation was reduced, and decreased cell proliferation and increased apoptosis were significantly rescued after the addition of the autophagy inhibitor autophinib. These consequences verify that, for cell proliferation and apoptosis, the regulation of Per2 depends on autophagy in OSCC. However, the specific regulatory mechanism requires further investigation.

In summary, this study demonstrates for the first time that Per2 could regulate autophagy in OSCC cells by relying on the PI3K/AKT/mTOR signaling pathway. Meanwhile, autophagy has a positive regulatory effect on apoptosis and a negative regulatory effect on proliferation. These results indicate that Per2 may be a valuable therapeutic marker for OSCC patients and suggest that overexpression of Per2, combined with activation of autophagy, may be a promising new approach to the treatment of OSCC.

## Supplementary Material

Supplementary tables.
