# Supplementary material for: Overexpression of the clock gene Per2 suppresses oral squamous cell carcinoma progression by activating autophagy via the PI3K/AKT/mTOR pathway
Source: J Cancer. 2020 Mar 26;11(12):3655–66. doi: 10.7150/jca.42771 (PMC7150464; doi:10.7150/jca.42771)
Supplement: Supplementary file 1 — Supplementary tables. [file jcav11p3655s1.pdf]

Supplementary Table S1 Sequence of RT-qPCR Primer

**Gene**	**Sequence (5' to 3')**
Per2 F	GGCTGTTGTGATGCGTATTCC
Per2 R	GCTCACGTCGCTCATCTTGC
GAPDH F	AGTGGTATTGTAGGTGCTGTGGTC
GAPDH R	ACGGTGTCGTAGCCTTCTGG

Supplementary Table S2 Information about Antibodies

**Antibody**	**Product Manufacturer**	**Code**
Anti-Per2	Abcam	ab179813
Anti-LC3B	Abcam	ab48394
Anti-SQSTM1/P62	CST	8025
Anti-Beclin1	CST	3495
Anti-PIK3CA	CST	4249
Anti-AKT	CST	4691
Anti-pAKT	CST	4060
Anti-mTOR	CST	2983
Anti-pmTOR	CST	5536
Anti-GAPDH	Proteintech	10494-1-AP
GoatAnti-Rabbit IgG	Proteintech	SA00001-2
